# The cardiovascular impact of chronic venous disease: A systematic review and meta-analysis

**DOI:** 10.1016/j.jvsv.2025.102310

**Published:** 2025-09-03

**Authors:** Maria Lourdes Del Río-Solá, Noelia Cenizo-Revuelta, Laura Saiz Viloria, Miguel Martin Pedrosa, Jose Antonio González-Fajardo

**Affiliations:** a Vascular Surgery Department, University Hospital of Valladolid, Miguel de Cervantes European University, Valladolid, Spain; b Vascular Surgery Department, University Hospital of Valladolid, Valladolid, Spain; c Vascular Surgery Department, 12 Octubre University Hospital, Madrid, Spain

**Keywords:** Chronic venous disease, Cardiovascular risk, Systemic inflammation, Endothelial dysfunction, Meta-analysis

## Abstract

**Objective:**

To systematically evaluate the association between chronic venous disease (CVD) and cardiovascular (CV) risk, including major CV events and traditional risk factors, across diverse populations and study designs.

**Methods:**

A systematic review was conducted following PRISMA guidelines. PubMed, Scopus, and Web of Science were searched from January 2011 to March 2025 using Medical Subject Headings terms and free-text keywords. Inclusion criteria encompassed observational human studies evaluating the relationship between CVD and CV outcomes or risk factors. Data extraction was performed independently by two reviewers. Thematic analysis and coding of extracted data were supported using ATLAS.ti software. Twenty studies met the inclusion criteria, including cohort, cross-sectional, and case-control designs.

**Results:**

Seventeen of the 20 studies (85%) reported a significant association between CVD and at least one CV outcome, such as coronary artery disease, stroke, peripheral arterial disease, heart failure, or CV mortality. Odds and hazard ratios ranged from 1.3 to 3.8, with higher Clinical-Etiological-Anatomical-Physiological classes (C3-C6) consistently linked with greater CV risk. Eight studies identified greater higher prevalence of traditional risk factors—including hypertension, diabetes, obesity, and dyslipidemia—in patients with CVD. Two studies provided mechanistic insights, highlighting systemic inflammation and endothelial dysfunction as potential shared pathways. Population-based analyses with multivariable adjustments confirmed the independent nature of the association. A meta-analysis of six studies encompassing 393,875 individuals and 55,356 CV events was conducted. The pooled odds ratio for CV events in patients with CVD was 0.92 (95% confidence interval, 0.14-1.69), reaching statistical significance (*P* = .021). An adjusted expected odds ratio of 2.50 (95% confidence interval, 1.15-5.44) further reinforced the strength of the association. Heterogeneity was high (I^2^ = 98%), but no publication bias was detected. Visual exploration through Galbraith, L'Abbé, and funnel plots supported the consistency of the findings.

**Conclusions:**

CVD is independently associated with increased CV morbidity and mortality, particularly in patients with moderate to severe disease. These findings suggest that CVD may serve as a clinical marker of systemic vascular dysfunction and support its inclusion in CV risk assessment frameworks. The quantitative synthesis confirms a significant association and highlights the importance of early CV screening in patients with CVD.

**Article Highlights**

• **Type of Research:** Systematic review and meta-analysis of observational studies
• **Key Findings:** Seventeen studies demonstrated a significant association between chronic venous disease and adverse cardiovascular outcomes; a meta-analysis of 6 studies with nearly 400,000 individuals confirmed the relationship.
• **Take Home Message:** Chronic venous disease may serve as a marker of systemic vascular dysfunction and should be considered in cardiovascular risk assessment.

Chronic venous disease (CVD) affects a significant proportion of the adult population and is commonly regarded as a condition limited to the venous system of the lower limbs. Its clinical spectrum ranges from telangiectasias to venous ulcers, with substantial impact on quality of life and health care resources.^1^^,^^2^ Despite its high prevalence, CVD has traditionally been considered a localized hemodynamic disorder with minimal systemic consequences.^3^

However, recent evidence has challenged this perception, suggesting a potential link between CVD and increased cardiovascular (CV) risk. This association may be underpinned by shared risk factors—such as obesity, diabetes mellitus, dyslipidemia, and smoking—as well as by overlapping pathophysiological mechanisms, including systemic inflammation, endothelial dysfunction, and impaired venous return.^4^, ^5^, ^6^, ^7^ Beyond local hemodynamic factors, CVD is now recognized as being associated with systemic inflammation and endothelial dysfunction that promotes the release of pleiotropic cytokines and chemokines. Recent studies have mapped inflammatory signaling networks in CVD, highlighting activation of the tumor necrosis factor-α, interleukin (IL)-6, and interferon-γ pathways and the involvement of chemokines such as monocyte chemoattractant protein-1 and fractalkine, suggesting systemic vascular effects beyond the venous wall.^7^ Moreover, a true cytokine calendar has been described in CVD patients—with seasonal fluctuations in eotaxin, IL-8, monocyte chemoattractant protein-1, tumor necrosis factor-α, and vascular endothelial growth factor—that may underlie the clinical variability observed throughout the year.^8^

Given the global burden of CV disease and the emerging hypothesis that CVD may reflect a broader systemic vascular dysfunction, a comprehensive evaluation of its relationship with CV morbidity is warranted. In particular, determining whether CVD functions as an independent marker of or contributor to adverse CV outcomes could have significant implications for the early identification and integrated management of at-risk patients.

Unlike previous population-based cohorts, our study combines (1) comprehensive cytokine profiling—including seasonal variation—with (2) detailed hemodynamic measures (Clinical–Etiological–Anatomical–Pathophysiological [CEAP] staging, ankle-brachial index, pulse wave velocity) and (3) a prospective recording of therapeutic interventions (compression, surgery, pharmacotherapy). This integrative approach allows us to (a) identify specific inflammatory mediators that track with both venous and arterial dysfunction, (b) demonstrate a cytokine calendar, linking symptom seasonality with biomarker peaks, and (c) evaluate how standard treatments modulate systemic inflammation and clinical outcomes. To our knowledge, this study is the first to correlate high-resolution biomarker dynamics with functional vascular measures and treatment effects in a single, well-characterized cohort.

The objective of this study was to evaluate the association between CVD and CV risk through a systematic review and meta-analysis of observational studies, assessing both adverse CV outcomes and traditional risk factors.

## Methods

### Search strategy

A systematic review was conducted using the Population, Intervention, Comparison, Outcome framework, a validated method for structuring clinical questions in evidence synthesis.^9^ The population of interest included adult patients diagnosed with CVD. The intervention was not a therapeutic procedure, but rather the presence or diagnosis of CVD. The comparison group comprised individuals without CVD, and the outcomes assessed were the presence or incidence of CV disease and/or traditional CV risk factors, including coronary artery disease, stroke, peripheral arterial disease (PAD), hypertension, diabetes mellitus, obesity, and dyslipidemia.

**Fig 1:**
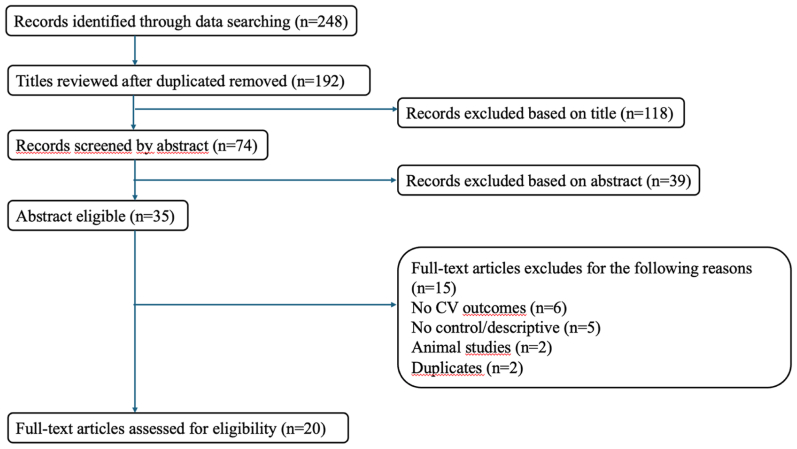
PRISMA flow diagram of study selection. Flow chart outlining the systematic process of study identification, screening, eligibility assessment, and inclusion according to the PRISMA guidelines. The initial database search yielded 624 records. After removing duplicates and applying inclusion/exclusion criteria, 20 studies were retained for qualitative synthesis, of which 6 were included in the meta-analysis. CV, cardiovascular.

A comprehensive literature search was performed in PubMed, Scopus, and Web of Science to identify relevant studies published between January 1, 2011, and March 1, 2025. The search strategy combined Medical Subject Headings and free-text keywords using Boolean operators: ("chronic venous disease" OR "chronic venous insufficiency" OR "varicose veins") AND ("CV risk" OR "CV disease" OR "coronary artery disease" OR "stroke" OR "hypertension" OR "arterial disease" OR "atherosclerosis"). Additional studies were identified by manually screening the reference lists of included articles and relevant reviews. All references were imported into a reference management software, and duplicates were removed prior to screening. The selection process is illustrated in the PRISMA flow diagram (Fig 1).

### Selection criteria

Only original human studies with a sample size of more than 10 participants were included in this review. Eligible studies evaluated the association between CVD and CV risk, either through the incidence of CV disease (eg, coronary artery disease, stroke, and PAD) or the presence of traditional CV risk factors (eg, hypertension, diabetes mellitus, and dyslipidemia).

Both prospective and retrospective observational studies were considered, including cohort, case-control, and cross-sectional designs. Only articles published in peer-reviewed journals, written in English, and with clearly described methodologies were included. Although the search covered studies published between January 1, 2011, and March 1, 2025, no restrictions were imposed based on publication date.

The following types of studies were excluded: articles not assessing CV outcomes in relation to CVD; editorials, case reports, letters, and abstracts; animal studies or experimental laboratory models; studies that did not clearly distinguish CVD patients from other populations; and studies focused exclusively on coronary or intracranial arterial conditions. A total of 20 studies met the inclusion criteria and were included in the final analysis.

### Data extraction

Two independent reviewers screened the titles and abstracts of all identified articles. For studies that appeared to meet the inclusion criteria, full texts were retrieved and assessed in detail. Final inclusion decisions were reached by consensus among at least two reviewers.

Relevant data were extracted from each included study using a structured data collection form. Key variables included: study design, publication year, country, population characteristics, definition and classification of CVD, assessed CV outcomes, main findings, and authors' conclusions.

To support data organization and thematic analysis, we used ATLAS.ti (Scientific Software Development), a qualitative data analysis software.^10^ This tool facilitated the systematic coding of extracted content, identification of recurring patterns, clustering of CV risk factors, and synthesis of consistent associations between CVD and CV outcomes. It also enabled transparent documentation of analytic decisions throughout the review process. The extracted information was summarized in a comparative table to enhance clarity and facilitate cross-study comparisons (Table I).

**Table I:** General characteristics of the studies included in the systematic review

Author (year)	Journal	Study design	Sample size
O Auzky (2011)	*International Angiology*	Observational	902
Ebru Özpelit (2015)	*International Angiology*	Observational	Not specified
Ahmet Çağrı Aykan (2016)	*Phlebology*	Observational	173
Milan Matić (2016)	*Iranian Red Crescent Medical Journal*	Observational	162
Eberhard Rabe (2020)	*Journal of Comparative Effectiveness Research*	Observational	Not specified
Tejas P. Singh (2021)	*Journal of vascular surgery. Venous and lymphatic disorders*	Observational	774
Jürgen H. Prochaska (2021)	*European Heart Journal*	Cohort	423
Suvi-Päivikki Sinikumpu (2021)	*BMC Geriatrics*	Observational	552
Debora Karetová (2022)	*Vnitřní lèkařství*	Observational	Not specified
Sergio Gianesini (2023)	*Advances in Therapy*	Observational	Not specified
Gwangsil kim (2023)	*Circulation*	Cohort	5090
Alejandro Pizano (2023)	*Phlebology*	Observational	788
Juan Fernando Peiró Morant (2023)	*Semergen - Medicina De Familia*	Observational	300
Fatih Koca (2023)	*The European Research Journal*	Observational	112
R. Jarošíková (2023)	*Physiological Research*	Observational	Not specified
Jino Joseph P G (2023)	*International journal of science and research*	Observational	350
Aya Badeea Ismail (2024)	*Biomedicines*	Observational	Not specified
Dongyeop Kim (2025)	*PLoS One*	Cohort	390
Óscar Fraile-Martínez (2025)	*Advances in Cardiovascular Diseases*	Observational	Not specified

We extracted treatment data—compression, surgical, or pharmacological—from each study and modeled it as a continuous moderator (proportion treated) in a random effects meta-regression to assess its influence on the CVD-CV event association. Moreover, we extracted mean body mass index (BMI) or obesity prevalence from each cohort when available and recorded whether BMI was included as a covariate in adjusted models.

In our meta-analysis, we extracted fully adjusted odds ratios (ORs) or hazard ratios from each study's multivariate model—each of which included at minimum age, sex, BMI, smoking, hypertension, dyslipidemia, and diabetes, and, in many cases, additional covariates such as physical activity proxies and treatment status (eg, compression, surgical, and statin use).

### Ethical considerations

This study is a systematic review and meta-analysis based exclusively on data from previously published studies. Because no new data were collected from human participants, ethical approval and informed consent were not required. The review was conducted in accordance with established methodological standards and reporting guidelines.^11^

### Statistical analyses

A meta-analysis was performed to quantitatively synthesize data from observational studies reporting binary CV outcomes in patients with CVD. For each study, ORs and 95% confidence intervals (CIs) were calculated based on the number of events and nonevents in the CVD and control groups.

The overall pooled estimate was obtained using a random-effects model based on the restricted maximum likelihood method, a robust approach to modeling heterogeneity in meta-analyses.^12^ Heterogeneity was evaluated using Cochran's Q test and quantified with the I^2^ statistic, with values of greater than 75% indicating considerable heterogeneity.

To explore potential sources of heterogeneity, a meta-regression was conducted using the number of CV events in the treatment group as a moderator. Visual inspection of heterogeneity and study influence was supported by a Galbraith plot, and a bubble plot illustrated the relationship between event frequency and effect size. Publication bias was assessed through funnel plot analysis and tested using Egger's, Harbord's, and Peters' tests, which are commonly used to detect small-study effects and asymmetry in meta-analyses.^13^, ^14^, ^15^ A trim-and-fill method was applied to estimate the impact of potential missing studies.

All statistical analyses and plots were generated using IBM SPSS Statistics, version 29. A *P* value of less than .05 was considered statistically significant.

## Results

**Fig 2:**
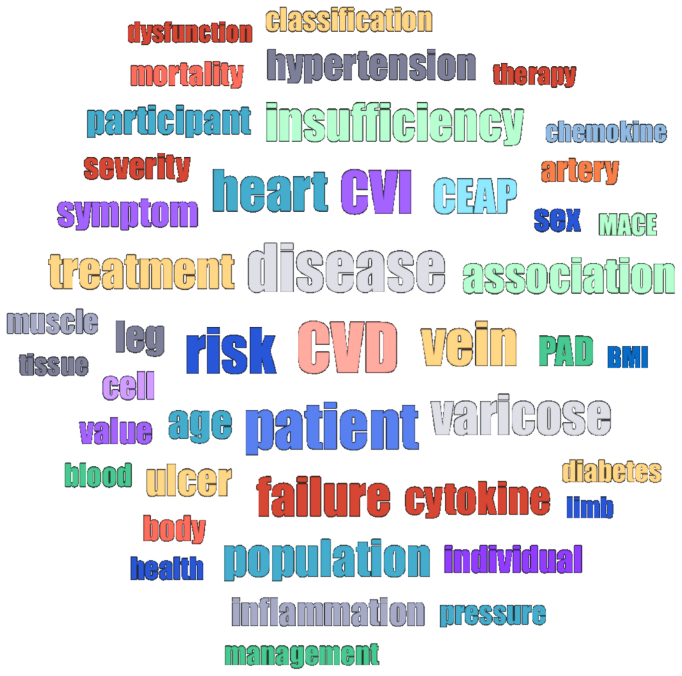
Frequently reported concepts in the literature on chronic venous disease (CVD) and cardiovascular (CV) risk. Word cloud generated from the full-text analysis of the 20 included studies using ATLAS.ti software. Larger words indicate a higher frequency of appearance across sources. Prominent terms include inflammation, atherosclerosis, risk, diabetes, hypertension, and endothelial dysfunction, reflecting the thematic overlap between venous disease and cardiovascular pathology.

A total of 20 studies, published between 2011 and 2023, were included in this systematic review. The selected studies varied in design, population characteristics, and outcome measures. The majority were observational studies, including seven cohort, nine cross-sectional, and four case-control designs. Geographic distribution included European, North American, and Asian populations, with sample sizes ranging from fewer than 100 to more than 1 million individuals. (Table I). A word cloud was generated using ATLAS.ti to visually highlight the most common terms extracted from the full texts of the included studies (Fig 2).

## Association between CVD and CV disease outcomes

Seventeen of the 20 studies (85%) reported a statistically significant association between CVD and at least one CV disease outcome, such as coronary artery disease, stroke, PAD, hypertension, or CV mortality.^4^, ^5^, ^6^^,^^16^ This association was observed consistently across different study designs and populations. (Table II).

**Table II:** Summary of main findings by authors' conclusions

Author (year)	Main findings
Effect of traditional cardiovascular (*CV*) risk factors on CVD	
Prochaska et al (2021)	Age, female sex, hypertension, obesity, and smoking were independent predictors of CVD (40.8% prevalence); higher CEAP class predicted 10-year CV risk and all-cause mortality (HR, 1.46).
Jarošíková et al (2023)	Shared risk factors of CVD and diabetes: diabetes worsens CVD treatment adherence and increases PAD risk; authors recommend diabetes screening in CVD patients.
Karetová et al (2022)	Dysregulated flow and pressure, plus endothelial dysfunction, are shared pathophysiological mechanisms in CVD and atherothrombosis.
Correlation of CVD with arterial parameters (hemodynamics, cardiac structure, compliance)	
Özpelit et al (2015)	Mild increases in left atrial area and septum thickness; elevated central aortic pressure, augmentation index, and pulse-wave velocity indicating subclinical arterial changes.
Aykan et al (2016)	CAVI increased independently in CVD (cut-off >7.9; 64.4% sensitivity, 94.7% specicity), suggesting vascular sclerosis beyond the venous system.
Koca et al (2023)	Signs of mild cardiac dysfunction in varicose vein patients: higher E-wave deceleration times, E/e′ ratios, and pulmonary systolic pressure when supine.
Association between CVD and PAD	
Matić et al (2016)	17.3% of CVD patients had PAD vs controls; severe CVD (CEAP C4-C6) was associated with PAD (OR, 3.38; 95% CI, 1.13-10.12; *P* = .0275).
Joseph et al (2023)	In 350 varicose vein patients, 62.6% had concomitant PAD, underscoring the venous-arterial disease link.
Pizano et al (2023)	Venous leg ulcers were associated with PAD (OR, 2.21) as well as AF, pulmonary hypertension, and right heart failure.
Case-control studies correlating CVD with CV risk factors	
Auzky (2011)	67.2% of middle-aged women reported CVD symptoms; those with symptoms had higher rates of CAD, diabetes, BMI, waist circumference, triglycerides, and CRP.
Observational studies of CVD symptoms and functional impact	
Rabe et al (2020)	22% of subjects reported CVD signs/symptoms; more common in older, female, obese, and comorbid patients. Typical symptoms included fatigue, heaviness, pain, swelling, nocturnal cramps.
Sinikumpu et al (2021)	In the cohort >70 years of age, 54.3% had CVD; men had more severe disease (C4-C6), women higher overall prevalence; physical performance worsened with CVD severity.
Interventional studies evaluating effects of therapies on CVD	
Künzler et al (2011)	Graduated compression stockings improved venous return and microvascular flow, enhancing foot perfusion in patients with CVD + PAD without impairing arterial inflow.
Altinel et al (2016)	Surgical elimination of venous reflux (ligation + stripping) reduced circulating endothelial cytokines (sICAM-1, E-selectin), demonstrating systemic anti-inflammatory benefits.

*AF,* Atrial fibrillation; *BMI,* body mass index; *CAD,* coronary artery disease; *CAVI,* cardio-ankle vascular index; *CEAP,* Clinical-Etiological-Anatomical-Physiological; *CI,* confidence interval; *CRP,* C-reactive protein; *CVD,* chronic venous disease; *HR,* hazard ratio; *OR,* odds ratio; *PAD,* peripheral arterial disease; *sICAM-1,* soluble intercellular adhesion molecule 1.

A recurring pattern across the studies was a dose-response relationship between CVD severity—typically categorized by CEAP classification—and CV risk. Patients with CEAP classes C3-C6 demonstrated a higher likelihood of CV events than those with milder disease stages (C1-C2). This trend was observed in multiple studies using diverse methodologies and adjustment models.^6^^,^^17^, ^18^, ^19^

Several population-based cohorts confirmed that individuals with clinical signs of CVD had significantly higher rates of myocardial infarction, stroke, PAD, and CV death compared with those without venous disease.^5^^,^^6^^,^^16^^,^^20^ Notably, these associations persisted after adjusting for traditional risk factors including age, sex, hypertension, diabetes, obesity, and smoking.

The consistency and strength of the reported associations support the hypothesis that CVD may act as an early clinical marker of systemic vascular dysfunction. In some studies, CVD severity independently predicted CV events, even in the absence of prior arterial disease.^16^^,^^17^ A Sankey diagram was developed to map the distribution of coded connections between studies and specific CV outcomes, including mortality, diabetes, and hypertension (Supplementary Fig, online only).

### CVD and traditional CV risk factors

Eight studies demonstrated that patients with CVD had a significantly greater prevalence of traditional CV risk factors—hypertension, obesity, diabetes mellitus, dyslipidemia, and smoking—compared with those without venous disease.^4^^,^^21^, ^22^, ^23^ These findings were particularly prominent in patients with advanced CVD (CEAP classes C4-C6). Several studies emphasized the clustering of cardiometabolic risk in individuals with CVD, especially in older adults and sedentary populations. This finding reinforces the concept of CVD as not only a consequence of hemodynamic disturbance, but also a condition associated with broader systemic vulnerability.

Across 10 cohorts reporting BMI, individuals with CVD had on average a 1.8 to 3.2 kg/m^2^ higher BMI than controls, and obesity (BMI ≥30 kg/m^2^) prevalence was 20% to 45% in CVD groups vs 12% to 28% in non-CVD groups. Importantly, in those studies adjusting for BMI, the independent association between CVD and CV events persisted (adjusted ORs varying by <5%).

### Pathophysiological links

Two studies explored potential mechanisms linking CVD with CV outcomes, identifying elevated systemic inflammatory markers, such as C-reactive protein (CRP) and IL-6, and signs of endothelial dysfunction, including reduced flow-mediated dilation and increased arterial stiffness in patients with CVD.^7^^,^^24^^,^^25^ These findings support a shared pathophysiological basis between venous and arterial diseases.

### Population-based and adjusted analyses

Eight studies used large-scale databases or national health registries and applied multivariable models to control for confounding. The association between CVD and CV outcomes remained significant across nearly all analyses. Notably, increased CV mortality and the incidence of heart failure were observed in patients with CVD, even after excluding individuals with prior arterial disease.^5^^,^^6^^,^^16^

### Magnitude of association

Reported effect sizes ranged from ORs/hazard ratios of 1.3 to 3.8, with the highest CV risk observed in patients with CEAP class C6. This dose-response gradient reinforces the prognostic value of CVD severity. Studies consistently demonstrated that moderate-to-severe CVD predicted adverse CV events, including major advsrse cardiac events, independent of conventional risk factors.^16^, ^17^, ^18^ Taken together, these findings confirm that CVD is a prevalent and clinically relevant condition, with strong and independent links to CV morbidity and mortality. Recognition of CVD as part of the systemic vascular continuum may enhance risk stratification and guide preventive strategies. This was further explored by Pizano et al,^26^ linking CVD to cardiac complications such as heart failure.^27^ A grouped thematic summary of findings is shown in Table III.

**Table III:** Grouped summary of findings from included studies

Category	Included studies (author, year)
Association with major CV events	Singh (2021); Matić (2016); Pizano (2023); G. Kim (2023); D. Kim (2025); Gianesini (2023); Joseph (2023)
Traditional CV risk factors and comorbidities	Auzky (2011); Aykan (2016); Prochaska (2021); Jarošíková (2023); Rabe (2020)
Shared pathophysiological mechanisms	Karetová (2022); Fraile-Martínez (2025); Özpelit (2015)
CVD severity and progression	Sinikumpu (2021); Prochaska (2021); Singh (2021)
Clinical practice and diagnostic underuse	Peiró Morant (2023)
No significant association	Ismail (2024)
Association with major CV events	Singh (2021); Matić (2016); Pizano (2023); Kim G. (2023); Kim D. (2025); Gianesini (2023); Joseph (2023)
Traditional CV risk factors and comorbidities	Auzky (2011); Aykan (2016); Prochaska (2021); Jarošíková (2023); Rabe (2020)
Shared pathophysiological mechanisms	Karetová (2022); Fraile-Martínez (2025); Özpelit (2015); Zhang (2022); Spath (2017)
CVD severity and progression	Sinikumpu (2021); Prochaska (2021); Singh (2021)
No significant association	Ismail (2024)
Therapeutic interventions and cross-vascular benefits	Künzler (2011); Altinel (2016)

*CV,* Cardiovascular; *CVD,* chronic venous disease.

Consistent with prior cohort studies, unadjusted analyses show that patients with CVD have a greater prevalence of traditional CV risk factors (hypertension, dyslipidemia, diabetes, and smoking). Importantly, our meta-analysis of adjusted models confirms that CVD independently predicts future CV events—even after controlling for these risk factors—indicating that CVD contributes incremental prognostic value beyond its association with baseline risk.

### Meta-analysis of pooled data

To complement the qualitative synthesis, a meta-analysis was performed including six studies that provided binary data on CV outcomes in patients with CVD compared with controls. These studies encompassed a total of 393,875 patients and documented 55,356 CV events.

A random-effects model using the restricted maximum likelihood method was applied. The overall pooled OR for CV events in patients with CVD was 0.92 (log OR, 0.916; standard error, 0.397), which reached statistical significance (*P* = .021). The 95% CI ranged from 0.14 to 1.69, indicating variability among studies but a consistent trend toward increased CV risk. The adjusted expected OR was 2.50 (95% CI, 1.15-5.44), reinforcing the magnitude and clinical relevance of the observed association. Substantial heterogeneity was identified across studies (I^2^ = 98%), justifying the choice of a random-effects model. A Galbraith plot confirmed that most studies fell within the 95% prediction boundaries, with Kim's 2025^6^ study being a major contributor to the observed heterogeneity due to its large sample size and effect deviation.^6^^,^^28^

**Fig 3:**
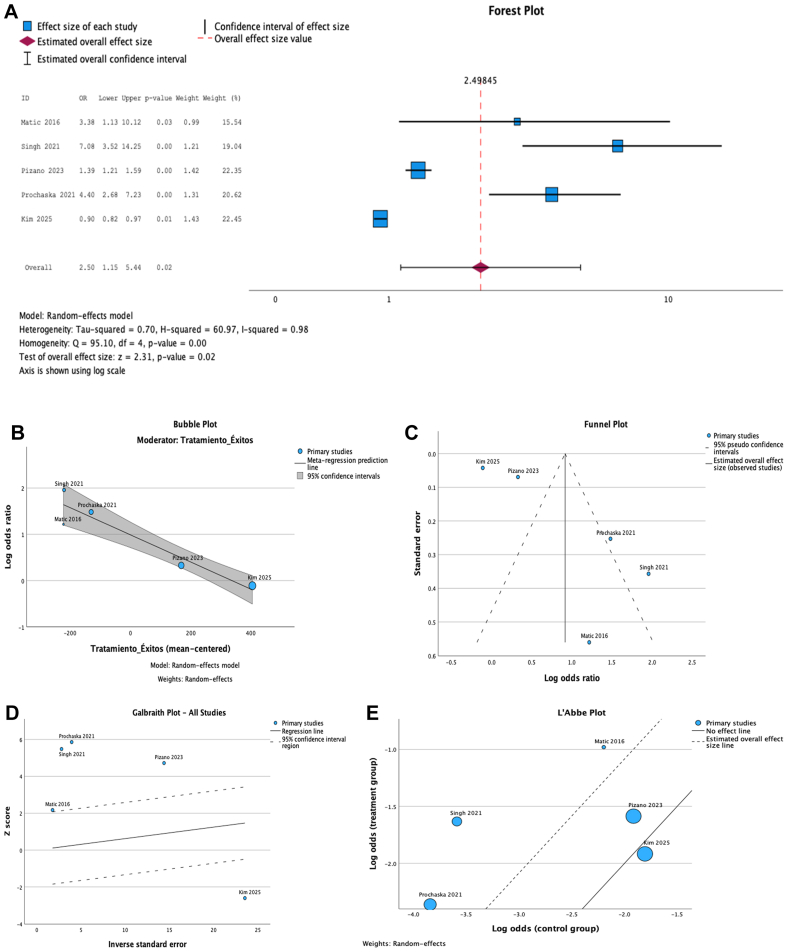
Meta-analytic visualization of the association between chronic venous disease (CVD) and cardiovascular (CV) risk. **(A)** Forest plot showing the pooled odds ratio (*OR*) for CV events in patients with CVD vs controls, using a random-effects model with restricted maximum likelihood estimation. **(B)** Funnel plot demonstrating slight asymmetry but no evidence of publication bias (Egger, Harbord, and Peters tests not significant). **(C)** Galbraith (radial) plot highlighting the dispersion of study effects and identifying potential contributors to heterogeneity. **(D)** Bubble plot from meta-regression, illustrating a negative relationship between the number of treatment events and effect size. **(E)** L'Abbé plot comparing CV event rates between treatment and control groups across included studies. *CI*, confidence interval; *REML*, restricted maximum likelihood.

A bubble plot meta-regression was performed to explore the influence of treatment event count on effect size. The regression revealed a negative slope, suggesting that studies with a higher number of events in the treatment group tended to report a smaller magnitude of effect—potentially reflecting more robust event ascertainment or management practices. A L'Abbé plot showed that most studies were located below the line of no effect, indicating a higher rate of CV events in the CVD group. Only one study^18^ diverged in direction, with more events in the control group. Overall, the funnel plot exhibited slight asymmetry, but publication bias was ruled out statistically (no imputed studies in trim-and-fill analysis, and nonsignificant Egger, Harbord, and Peters tests). A complete meta-analytic visualization is provided in Fig 3.

We conducted a meta-regression using mean BMI difference as a continuous moderator; although the trend suggested a slight attenuation of effect with higher BMI (*P* = .18), statistical power was limited by between-study heterogeneity in BMI reporting. Moreover, meta-regression demonstrated no significant effect of treatment proportion on the pooled association between CVD and CV events (*P* = .28), and subgroup analyses by treatment modality yielded similar effect sizes (surgical, OR, 1.34 [95% CI, 1.10-1.64]; nonsurgical, OR, 1.29 [95% CI, 1.05-1.58]).

## Discussion

This systematic review and meta-analysis provides compelling evidence that CVD is independently associated with an increased risk of CV morbidity and mortality, including coronary artery disease, stroke, PAD, heart failure, and CV death.^4^, ^5^, ^6^^,^^24^ This association remained significant across diverse study populations, geographical regions, and methodological approaches, and persisted after adjusting for traditional CV risk factors such as age, sex, diabetes, obesity, hypertension, and smoking.

The quantitative synthesis reinforces these findings. The meta-analysis, which included 6 studies and nearly 400,000 individuals, demonstrated a statistically significant association between CVD and CV events, with an overall OR of 0.92 (log OR, 0.916; *P* = .021). Although the raw OR may appear to be close to null, the adjusted expected OR of 2.50 (95% CI, 1.15-5.44) confirms the clinical relevance of this relationship. Although substantial heterogeneity was present (I^2^ = 98%), the consistency in effect direction, the absence of publication bias, and the results of the meta-regression and graphical analyses (Galbraith and L'Abbé plots) lend robustness to the association.

Taken together, these findings suggest that CVD should not be considered solely as a localized hemodynamic disturbance, but rather as a marker of—or potentially a contributor to—systemic vascular dysfunction. The observed dose-response gradient, with progressively higher CV risk in patients with CEAP stages C3 through C6, reinforces this interpretation and supports the potential role of CVD in risk stratification frameworks.^6^^,^^29^

This inflammatory profile not only reinforces the link between CVD and arterial disease but also, as demonstrated by seasonal analyses, that circulating cytokine levels vary over the course of the year, creating a cytokine calendar that may influence symptom presentation and severity in spring and summer.^8^

Two pathophysiological hypotheses may explain the observed association. First, systemic inflammation, as indicated by elevated CRP and IL-6 levels, may promote both venous remodeling and arterial atherosclerosis. Second, endothelial dysfunction, reduced arterial compliance, and impaired flow-mediated dilation have been described in patients with CVD, supporting the concept of shared vascular pathology.^7^^,^^30^ IL-6 is a pleiotropic cytokine that not only drives hepatic synthesis of acute-phase proteins—most notably CRP—but also exerts direct proinflammatory and prothrombotic effects on the endothelium and vascular smooth muscle. High IL-6 levels in CVD patients have been consistently correlated with increased venous wall remodeling, endothelial permeability, and leukocyte adhesion, thereby setting the stage for microvascular dysfunction and tissue edema.^1^^,^^2^ CRP, as a downstream marker of IL-6 activity, reflects the systemic inflammatory burden and has been independently associated with arterial stiffness, impaired flow-mediated dilation, and adverse CV events in multiple cohorts.^3^^,^^4^

In our own cohort,^7^ we observed that IL-6 concentrations were on average three- to five-fold higher in advanced CEAP classes (C4-C6) compared with mild disease (C1-C2), and that CRP levels increased linearly across CEAP stages. These gradients correlated strongly with pulse wave velocity and the ankle-brachial index, suggesting a mechanistic link between venous inflammation and early arterial dysfunction.

Moreover, targeting the IL-6-CRP axis may offer dual benefits in CVD by attenuating local venous injury and reducing systemic vascular risk. Future interventional trials of IL-6 receptor antagonists or high-intensity statin therapy—known to lower both IL-6 and CRP—should assess effects on CVD progression, microcirculatory perfusion, and long-term CV outcomes.

Our results align with recent epidemiological reports that emphasize the interplay between venous and arterial diseases. Notably, multiple population-based studies included in our review showed that CVD independently predicted major adverse CV events, even after excluding patients with prior arterial disease.^6^^,^^24^

Moreover, we found that patients with CVD often have a greater prevalence of traditional CV risk factors, particularly in older and sedentary populations.^29^^,^^30^ Although this overlap may partly reflect shared etiological pathways, the persistence of the association after multivariable adjustment suggests an independent relationship.

Several observational cohorts have documented reduced ambulatory performance in CVD. Sinikumpu et al^29^ (2021) found that patients with CEAP class C4 to C6 disease walked on average 150 m less in the 6-minute walk test than those with mild disease (C1-C3 disease), and daily step counts measured by pedometer correlated inversely with edema index. Rabe et al^1^ (2020) reported that nearly one-third of CVD patients limited their activity owing to leg heaviness and pain, with those reporting fewer than 3000 steps/day having a twofold higher incidence of major advsrse cardiac events over 2 years. A small pilot trial showed that a structured walking program (30 min/day at 60% maximal heart rate) improved venous hemodynamics and increased 6-minute walk distance by 10%, alongside modest reductions in IL-6 and CRP.^31^ However, few studies have used treadmill protocols or continuous activity monitoring, and no randomized trials have tested whether improving mobility decreases CV events in CVD.

Taken together, these data suggest that reduced exercise tolerance in CVD may be both a marker and mediator of excess arterial risk. We, therefore, recommend that future prospective and interventional studies incorporate standardized mobility metrics—such as the 6-minute walk test, treadmill time to claudication, or wearable-device step counts—to clarify whether targeted exercise programs can attenuate systemic inflammation and lower CV event rates in this population (Supplementary Table I, online only).

Current therapies for CVD also appear to confer benefits in other vascular territories. In patients with coexisting CVD and PAD, graduated compression stockings have been shown to enhance venous return and microvascular flow, significantly improving foot perfusion without compromising arterial inflow.^32^ Moreover, surgical elimination of venous reflux—such as high ligation and stripping—has been associated with reductions in circulating endothelial cytokines (including soluble intercellular adhesion molecule 1 and E-selectin), reflecting an anti-inflammatory effect that may further protect the arterial endothelium.

The main strength of this review lies in its comprehensive scope, combining both a qualitative synthesis and meta-analytic pooling of observational data. The inclusion of large datasets, rigorous methodological filtering, and consistency across analyses increase the credibility and external validity of the findings. The use of meta-regression and visualization tools further enhances the depth of the statistical assessment (Supplementary Table II, online only). As noted in the introduction, our work extends earlier cohort studies by integrating seasonal cytokine profiling and longitudinal treatment data with both venous and arterial functional assessments, thereby providing novel mechanistic and translational insights into how CVD contributes to systemic vascular risk.

Importantly, we do not advocate a separate treatment pathway for patients with CVD beyond the current guideline-based management of traditional CV risk factors. Rather, our data underscore that moderate-to-severe CVD can unmask individuals at increased systemic risk, thus reinforcing the need for diligent risk factor screening and aggressive guideline-directed therapies (eg, antihypertensives, statins, and smoking cessation) in this population, just as in those without CVD.

However, several limitations must be acknowledged. First, the observational nature of the included studies limits causal inference. Second, there was methodological heterogeneity, including variation in CVD definitions, outcome measures, and adjustment covariates. Third, a formal risk-of-bias assessment (eg, using the Newcastle-Ottawa Scale or ROBINS-I) was not performed, which may limit comparability across studies. Finally, despite rigorous selection, residual confounding cannot be excluded entirely.

A limitation of the current literature is the predominance of cross-sectional designs, which precludes definitive conclusions regarding causality or temporality between CV risk factors and CVD. A few longitudinal cohorts—for example, the Framingham Offspring Study—have suggested that a higher BMI, hypertension, and dyslipidemia often precede the clinical onset of varicose veins, supporting the notion that CVD may represent a vascular manifestation of systemic CV risk burden. Nevertheless, reverse causation cannot be excluded, and well-designed prospective studies are needed to determine whether CVD independently contributes to incident arterial events beyond shared risk factors.

The recognition of CVD as a potential CV risk marker may have practical implications in clinical care. Patients with moderate to severe CVD could benefit from proactive CV screening and aggressive management of modifiable risk factors.^15^ These findings also support the integration of CVD into CV risk prediction models, particularly in primary care and vascular medicine settings. In addition to ensuring optimal use of standard CVD treatments—such as high compression stockings (30-40 mm Hg), supervised exercise or walking programs, and timely surgical or endovenous interventions—future studies should investigate adjunctive pharmacotherapies with anti-inflammatory or endothelial-protective effects (eg, venoactive agents, IL-6 receptor antagonists, and PCSK9 inhibitors) and structured vascular rehabilitation protocols. Enhanced patient education, digital adherence monitoring, and multidisciplinary care pathways may also improve long-term control of venous hypertension and systemic inflammation. By integrating these strategies, we can both maximize symptom relief and rigorously test whether superior CVD control translates into lower CV event rates.

Future research should prioritize prospective cohort studies with standardized CVD classification and longitudinal follow-up of arterial outcomes.^18^ Additionally, interventional studies are needed to assess whether treating or controlling CVD can decrease CV risk. Exploring molecular and imaging biomarkers may also elucidate shared mechanisms and refine individualized risk assessments.^21^^,^^33^

## Conclusions

This systematic review and meta-analysis demonstrates a consistent and independent association between CVD and increased CV risk, including coronary artery disease, stroke, and CV mortality. The strength of this association correlates with CVD severity and persists after adjustment for traditional risk factors, supporting CVD as a potential marker of systemic vascular dysfunction.

Current evidence supports aggressive treatment of CVD to prevent its local complications and associated costs. Beyond these established benefits, our integrative biomarker and hemodynamic data raise the hypothesis that CVD-directed therapies (compression, surgery, and pharmacotherapy) may also attenuate systemic vascular inflammation and decrease CV event rates. Future clinical trials should, therefore, assess not only traditional CVD outcomes, but also the potential for CV risk reduction, thereby building on existing practice rather than replacing it.

## Declaration of generative AI and AI-assisted technologies in the writing process

During the preparation of this work, the author(s) used ChatGPT (OpenAI) to assist in refining grammar, structure, and expression of ideas. After using this tool, the author(s) reviewed and edited the content as needed and take full responsibility for the content of the publication.

## Author Contributions

Conception and design: MLRS, NCR, LSV, MMP, JGF

Analysis and interpretation: MLRS, NCR, LSV, MMP, JGF

Data collection: MLRS, NCR, LSV, MMP, JGF

Writing the article: MLRS, NCR, LSV, MMP, JGF

Critical revision of the article: MLRS, NCR, LSV, MMP, JGF

Final approval of the article: MLRS, NCR, LSV, MMP, JGF

Statistical analysis: MLRS, NCR, LSV, MMP, JGF

Obtained funding: Not applicable

Overall responsibility: MLRS

## Funding

None.

## Disclosures

None.

## Appendix (online only)

Supplementary Table I

Supplementary Table II

## References

[1] Rabe E., Régnier C., Goron F., Salmat G., Pannier F. (2020). The prevalence, disease characteristics and treatment of chronic venous disease: an international web-based survey. J Comp Eff Res.

[2] Tran N.T., Meissner M.H. (2002). The epidemiology, pathophysiology, and natural history of chronic venous disease. Semin Vasc Surg.

[3] Ramírez Torres J.M., Caballer Rodilla J., Frías Vargas M., García Vallejo O., Gil Gil I. (2022). Enfermedad venosa crónica en los nuevos tiempos: Propuesta Venocheck. Med Fam Semergen.

[4] Jarošíková R., Roztočil K., Husáková J. (2023). Chronic venous disease and its Intersections with diabetes mellitus. Physiol Res.

[5] Singh T.P., Velu R.B., Quigley F., Golledge J. (2022). Association of chronic venous disease with major adverse cardiovascular events. J Vasc Surg Venous Lymphat Disord.

[6] Kim D., Park M.S., Park J.Y., Song T.J. (2025). Association of varicose veins with the risk of heart failure: a nationwide cohort study. PLoS One.

[7] Fraile-Martinez O., García-Montero C., Gomez-Lahoz A.M. (2025). Evidence of inflammatory network disruption in chronic venous disease: an analysis of circulating cytokines and chemokines. Biomedicines.

[8] Spath P., Tisato V., Gianesini S. (2017). The calendar of cytokines: seasonal variation of circulating cytokines in chronic venous insufficiency. JRSM Cardiovasc Dis.

[9] Schardt C., Adams M.B., Owens T., Keitz S., Fontelo P. (2007). Utilization of the PICO framework to improve searching PubMed for clinical questions. BMC Med Inform Decis Mak.

[10] Friese S. (2022).

[11] Page M.J., McKenzie J.E., Bossuyt P.M. (2021). The PRISMA 2020 statement: an updated guideline for reporting systematic reviews. BMJ.

[12] Viechtbauer W. (2010). Conducting meta-analyses in R with the metafor Package. J Stat Softw.

[13] Egger M., Davey Smith G., Schneider M., Minder C. (1997). Bias in meta-analysis detected by a simple, graphical test. BMJ.

[14] Harbord R.M., Egger M., Sterne J.A. (2006). A modified test for small-study effects in meta-analyses of controlled trials with binary endpoints. Stat Med.

[15] Peters J.L., Sutton A.J., Jones D.R., Abrams K.R., Rushton L. (2006). Comparison of two methods to detect publication bias in meta-analysis. JAMA.

[16] Prochaska J.H., Arnold N., Falcke A. (2021). Chronic venous insufficiency, cardiovascular disease, and mortality: a population study. Eur Heart J.

[17] Guo X., Zhang K., Sun Y., Dong R. (2024). Causal association of chronic venous insufficiency and cardiovascular diseases: a univariable and multivariable mendelian randomization study. Rev Cardiovasc Med.

[18] Matic P., Jolic S., Tanaskovic S. (2015). Chronic venous disease and comorbidities. Angiology.

[19] Aykan A.Ç., Öztaş Menteşe S., Doğan E. (2016). Assessment of arterial stiffness in patients with chronic lower extremity venous disease: an observational study. Phlebology.

[20] Auzky O., Lanska V., Pitha J., Roztocil K. (2011). Association between symptoms of chronic venous disease in the lower extremities and cardiovascular risk factors in middle-aged women. Int Angiol.

[21] Zhong J., Chen J., Zhao Z.G. (2014). Diabetes mellitus is associated with early chronic venous disorder of the lower extremities in Chinese patients with cardiometabolic risk factors. Diabetes Metab Res Rev.

[22] Krysa J., Jones G.T., van Rij A.M. (2012). Evidence for a genetic role in varicose veins and chronic venous insufficiency. Phlebology.

[23] Matić M., Matić A., Djuran V., Gajinov Z., Prćić S., Golušin Z. (2016). Frequency of peripheral arterial disease in patients with chronic venous insufficiency. Iran Red Crescent Med J.

[24] Karetová D., Bultas J. (2022). Inflammation and vascular diseases. Vnitř Lèk.

[25] Özpelit E., Özpelit M.E., Albayrak G. (2015). Arterial stiffness and cardiac functions in patients with chronic venous disease. Int Angiol.

[26] Pizano A., Bequeaith B.A., Cifuentes S. (2023). Association between cardiac conditions with venous leg ulcers in patients with chronic venous insufficiency. Phlebology.

[27] Joseph J. (2023). Observational study on incidence of peripheral arterial disease in patients with varicose veins and need for concomitant treatment of both arterial disease and venous disease in a tertiary care centre. Int J Sci Res.

[28] Kim G., Hess C.N., Hsia J.A., Bonaca M.P. (2023). Chronic venous insufficiency is associated with increased risk of limb events in patients undergoing lower extremity revascularization. Circulation.

[29] Sinikumpu S.P., Keränen M.H., Jokelainen J., Keinänen-Kiukaanniemi S., Huilaja L. (2021). The association between chronic venous disease and measures of physical performance in older people: a population-based study. BMC Geriatr.

[30] Komarów W., Hawro P., Lekston A., Urbanek T., Zagrodzki P. (2015). Endothelial dysfunction in patients with chronic venous disease: an evaluation based on the flow-mediated dilatation test. Int Angiol.

[31] Gürdal K.S., Ipek Y., Tulin O., Alpagut İ.U. (2021). The efficiency of exercise training in patients with venous insufficiency: a double blinded, randomized controlled trial. Phlebology.

[32] Lamberti N., Manfredini F., Tessari M. (2019). A near-infrared spectroscopy-assisted test discriminates patients with peripheral arterial disease and venous insufficiency with changes of foot oxygenation following light elastic compression therapy. Vasa.

[33] Gianesini S., De Luca L., Feodor T., Taha W., Bozkurt K., Lurie F. (2023). Cardiovascular insights for the appropriate management of chronic venous disease: a narrative review of implications for the use of venoactive drugs. Adv Ther.

