# Supplementary material for: The cardiovascular impact of chronic venous disease: A systematic review and meta-analysis
Source: J Vasc Surg Venous Lymphat Disord. 2025 Sep 3;14(1):102310. doi: 10.1016/j.jvsv.2025.102310 (PMC12826958; doi:10.1016/j.jvsv.2025.102310)
Supplement: Supplementary Table I [file mmc1.docx]

**Suplementary Table S1. Prospective Studies of CV Risk–Factor Interventions and Their Effects on CVD**

| **Study (Year)** | **Design** | **Intervention (Risk Factor)** | **CVD Outcome Measure** | **Main Findings** |
| --- | --- | --- | --- | --- |
| Framingham Offspring Study (2002) | Prospective cohort | Observational (BMI, HTN, lipids) | Incident varicose veins | Higher baseline BMI, hypertension, and dyslipidemia independently predicted new-onset CVD over 12 years of follow-up. |
| Pilot weight-loss programme (Martínez et al., 2018) | Single-arm interventional | Lifestyle (diet + exercise) | Leg symptom score; CEAP clinical stage | 8% mean weight loss led to a 25% reduction in leg heaviness scores and one‐stage improvement in CEAP class at 6 months. |
| Statin therapy observational study (Lee et al., 2020) | Prospective observational | High-intensity statins | Lower-limb edema volume; cytokine levels | Statin users showed a 15% decrease in tibial vein diameter and 20% lower IL-6 and CRP levels versus non-users at 1 year. |
| Antihypertensive trial (Garcia-Ruiz et al., 2021) | Randomized pilot (n = 60) | ACE inhibitor (ramipril) | Microvascular flow (laser Doppler); symptoms | Ramipril arm experienced 30% increase in microvascular perfusion and 18% reduction in pain score compared to placebo. |
| PCSK9-inhibitor observational cohort (Wang et al., 2023) | Prospective observational | PCSK9 monoclonal antibody | Venous reflux index; CEAP progression | PCSK9i users had stabilization of venous reflux measurements and no CEAP progression over 18 months, versus 12% in controls. |
