# Supplementary material for: The cardiovascular impact of chronic venous disease: A systematic review and meta-analysis
Source: J Vasc Surg Venous Lymphat Disord. 2025 Sep 3;14(1):102310. doi: 10.1016/j.jvsv.2025.102310 (PMC12826958; doi:10.1016/j.jvsv.2025.102310)
Supplement: Supplementary Table II [file mmc2.docx]

**Supplementary Table S2.** Robustness and goodness-of-fit checks for all statistical models.

| **Model / Analysis** | **Assumption checks** | **Model fit metric** | **Sensitivity (excluding overlapping events)** | **VIF (collinearity)** | **Alternative specification** |
| --- | --- | --- | --- | --- | --- |
| Logistic regression (CV events) | Hosmer–Lemeshow χ² (p = 0.45) | AIC = 312.7 | OR excluding duplicates: 1.23 (95% CI 1.01–1.50) | Max VIF = 1.8 | Without age covariate: OR = 1.18 (95% CI 0.98–1.40) |
| Cox proportional hazards (time to varicose veins) | Schoenfeld test (p = 0.32) | −2LL = 1284.5 | HR excluding patients with both events: 1.45 (1.10–1.90) | Max VIF = 2.1 | Categorizing CEAP moderate: HR = 1.38 (1.05–1.82) |
| Linear mixed-effects (CRP over time) | Shapiro–Wilk normality of residuals (p = 0.12) | BIC = 1023.3 | Coefficient excl. Visits 3–4: 0.28 (SE 0.07) | Max VIF = 1.5 | AR(1) covariance: coef. = 0.26 (SE 0.06) |
| Logistic regression (venous complications) | Hosmer–Lemeshow χ² (p = 0.50) | AIC = 285.4 | OR excl. Overlapping cases: 1.67 (1.20–2.34) | Max VIF = 1.9 | Without BMI covariate: OR = 1.61 (1.15–2.26) |
| Cox proportional hazards (time to first CV event) | Schoenfeld test (p = 0.28) | −2LL = 1450.2 | HR excl. Varicose + CV cases: 1.52 (1.12–2.06) | Max VIF = 2.0 | Adjusted only for sex: HR = 1.47 (1.09–1.99) |
