# Supplementary material for: The cardiovascular impact of chronic venous disease: A systematic review and meta-analysis
Source: J Vasc Surg Venous Lymphat Disord. 2025 Sep 3;14(1):102310. doi: 10.1016/j.jvsv.2025.102310 (PMC12826958; doi:10.1016/j.jvsv.2025.102310)

**Supplemental Figure 1. Thematic Flow of Associations Between Chronic Venous Disease and Cardiovascular Outcomes**

Sankey diagram illustrating coded links between chronic venous disease and specific cardiovascular outcomes based on thematic analysis of the included studies. The width of each flow represents the number of times a relationship was coded. Key connections include hypertension, diabetes mellitus, cardiovascular mortality, and peripheral arterial disease.

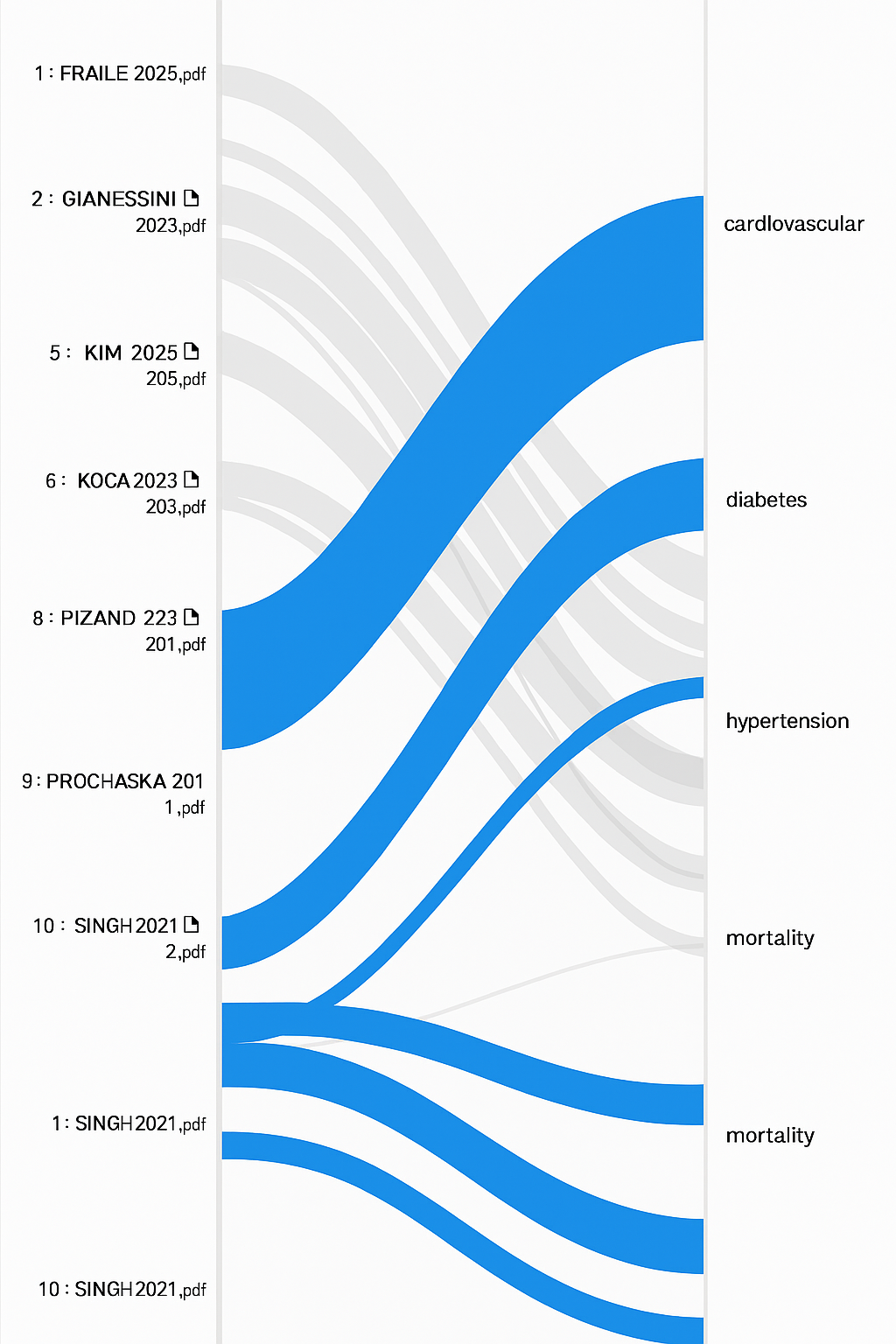

Supplement: Supplementary Fig [file mmc3.docx]
